# Complete plastid genome characterization and phylogenetic analysis of *Pentasachme caudatum* Wallich ex Wight (Gentianales: Apocynaceae) from Guangdong, China

**DOI:** 10.1080/23802359.2021.1885319

**Published:** 2021-03-15

**Authors:** Shenyu Miao, Yuqian Luo, Mary Ann C. Bautista, Tao Chen

**Affiliations:** a School of Life Sciences, Guangzhou University, Guangzhou, China; b Fairy Lake Botanical Garden, Chinese Academy of Sciences, Shenzhen, China; c South China Botanical Garden, Chinese Academy of Sciences, Guangzhou, China; d Graduate School, University of Chinese Academy of Sciences, Beijing, China

**Keywords:** Apocynaceae, Asclepiadoideae, Pentasachme caudatum, phylogeny, plastid genome

## Abstract

*Pentasachme caudatum* Wallich ex Wight is considered as one of the Asian enigmatic genera classified in the Asclepiadoideae (Apocynaceae). To determine its evolutionary relationship in the family, we sequenced and characterized the complete chloroplast genome of *P. caudatum*. The plastid genome of *P. caudatum* is 158,487 bp in length, containing a large single-copy (90,380 bp), a small single-copy (18,585 bp), and a pair of inverted repeats (24,761 bp). It has 127 annotated genes, consisting of 83 protein-coding, eight rRNA and 36 tRNA genes. Phylogenetic analysis using 76 protein-coding regions of the plastid genomes of related taxa showed that *P. caudatum* was resolved in a fully supported clade with *Orthanthera albida*. The newly sequenced *P. caudatum* provides essential genetic information that is useful for future phylogenetic studies in the family Apocynaceae.

In the Apocynaceae, the Asclepiadoideae has been established as a monophyletic group, however, systematic positions of the Asian genera like *Pentasachme* is still uncertain (Surveswaran et al. 2014). *Pentasachme* are characterized as rheophytic herbs, with slender, elliptic leaves and sessile inflorescences that bear few flowers with long, slender corolla (Surveswaran et al. 2014). In China, only one species of *Pentasachme* is recognized, *Pentasachme caudatum* Wallich ex Wight (Li et al. 1995). Morphologically, *P. caudatum* is easily confused with *Cynanchum stauntonii*, which complicates species identification (Li et al. 1995). Both species are considered medicinal, but they are used to treat different diseases. Hence, misidentifications for use as an herbal medicine may lead to potential safety risks. Authentication of the plant material is the initial and most important step in conducting either taxonomic or pharmacological studies. Thus, this study aimed to provide the complete plastid sequence of *P. caudatum* to be used as a genetic resource for future plant authentication.

Total DNA was extracted from fresh leaves of *Pentasachme caudatum* (2020061510, SZG, Tao Chen, taochen@szbg.ac.cn) collected from Sanzhoutian, Yantian District, Shenzhen, Guangdong, China (E114°16′09ʺ, N22°37′39ʺ) using a modified CTAB method (Murray and Thompson 1980). Sequencing was performed on the Illumina Novseq 600 platform producing paired-end sequences with an average read length of 150 bp and average sequencing depth of 2,369. Using the NGS-QC toolkit (Patel and Jain 2012), the raw Illumina reads were filtered and the resulting high quality reads were assembled via the *de novo* assembler SPAdes 3.11.0 (Vasilinetc et al. 2015). The assembled cp genome was annotated using Plann software (Huang and Cronk 2015), RNAmmer 1.2 Server (Lagesen et al. 2007) and tRNAscan-SE (Chan and Lowe 2019) for protein-coding genes, rRNA and tRNA genes. *Oncinotis tenuiloba* (KJ953908.1) was used as a reference in the plastid genome annotation.

The complete plastid sequence of *P. caudatum* has a total length of 158,487 bp and exhibit a typical quadripartite structure containing a large single copy region (90,380 bp) and a short single copy region (18,585 bp) separated by two inverted repeat regions (24,761 bp). The genome has 127 annotated genes, including 83 protein-coding, eight rRNA and 36 tRNA genes. Similar to other Asclepiadoideae plastid genomes, there are 17 genes with introns, 15 genes (*trn*K-UUU, *rps*16, *trn*G-UCC, *atp*F, *rpo*C1, *trn*L-UAA, *trn*V-UAC, *pe*tB, *pet*D, *rpl*16, *rpl*2, *ndh*B, *trn*I-GAU, *trn*A-UGC, *ndh*A) have a single intron, whilst *clp*P and *ycf*3 have two introns. The *rps*12 gene was also transpliced, with the 5′ exon located in the LSC region and 3′ exon duplicated in the IR regions. The overall GC content of the genome was 37.8%.

Phylogenetic position of *P. caudatum* was determined by constructing a Maximum Likelihood tree using RAxML 8.2.11 (Stamatakis 2014). The *P. caudatum* cp genome sequence was aligned with 19 other sequences from the Apocynaceae and ML tree was inferred using the GTR + I + G nucleotide substitution model with 1,000 replicates. *Gentiana* was designated as the outgroup. The resulting ML tree (Figure 1) showed that *P. caudatum* occupies a sister position to *Orthanthera albida*. The tree also confirms the sister tribe relationship between Ceropegieae (*P. caudatum, O. albida*) and Marsdenieae (*Marsdenia astephanoides, Hoya liangii, H. potsii*) (Endress et al. 2007; Surveswaran et al. 2014). Both tribes are characterized by having erect pollinaria (Endress et al. 2007). Moreover, this phylogenetic tree based on plastid genomes further supports the monophyly of the subfamily Asclepiadoideae and its close relationship to Secamonoideae.

**Figure 1.**
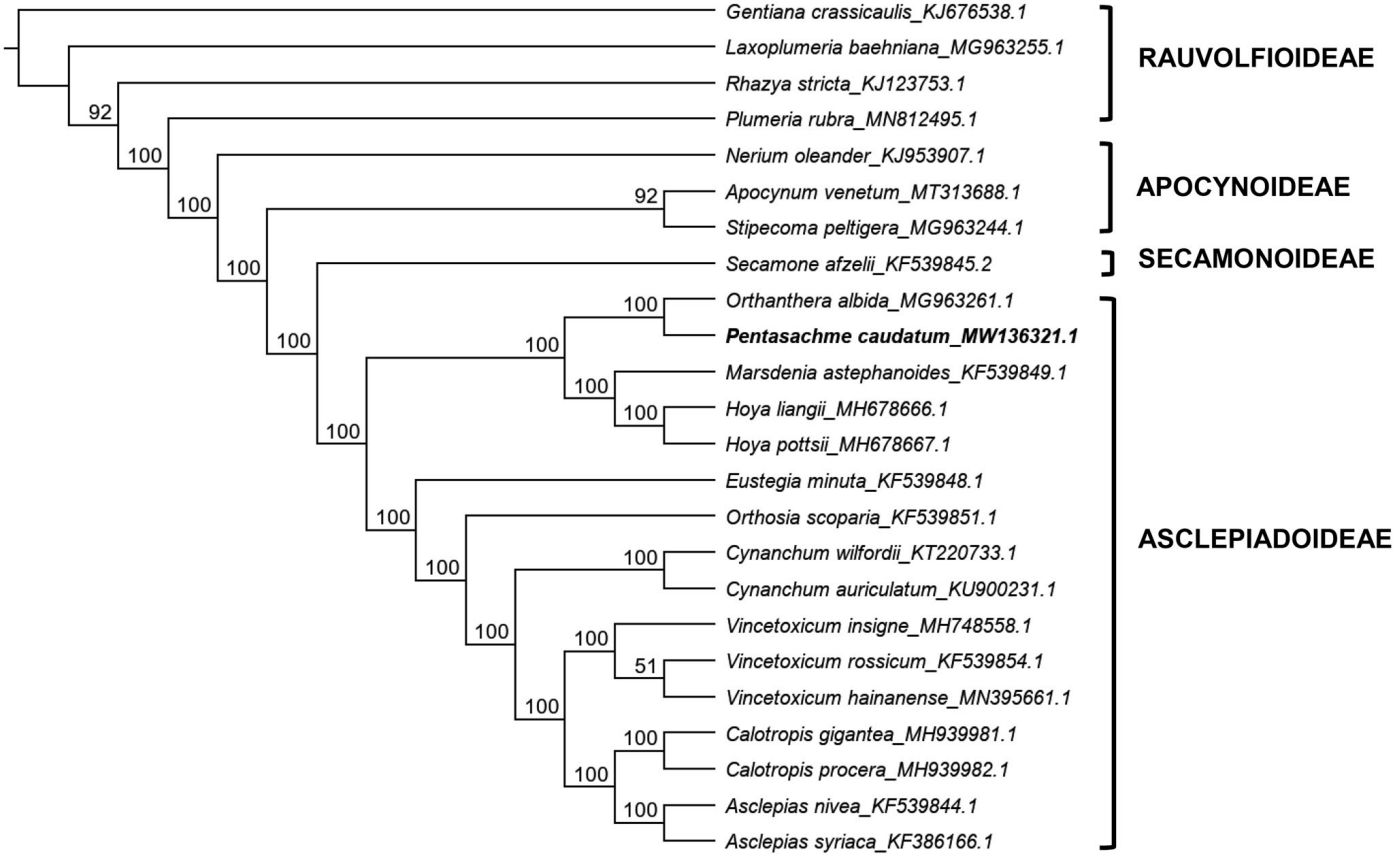
Maximum-likelihood tree showing phylogenetic placement of *P. caudatum* within the Apocynaceae. Bootstrap replicates based on 1000 iterations are indicated at the nodes and GenBank accession numbers follow the binomials.

## Data Availability

The complete chloroplast genome sequence of *Pentasachme caudatum* was deposited in NCBI GenBank with the accession number MW136321. Raw sequencing data were also available in SRA database with the BioSample number SAMN17080906, SRA number SRX9685746 and BioProject accession number PRJNA672143. (https://www.ncbi.nlm.nih.gov/nuccore/MW136321; https://www.ncbi.nlm.nih.gov/sra/PRJNA672143)
